# Morselized Femoral Head Impaction Bone Grafting of Large Defects in Ankle and Hindfoot Fusions

**DOI:** 10.1177/10711007241310411

**Published:** 2025-01-27

**Authors:** Tim Clough, Bakur Jamjoom, Naeem Jagani, Jared Quarcoopome, Rajesh Kakwani, David Townshend, Nicholas Cullen, Shelain Patel, Karan Malhotra, Matthew Welck

**Affiliations:** 1 Wrightington Hospital, Wigan, Lancashire, United Kingdom; 2 Royal National Orthopaedic Hospital NHS Trust, Foot & Ankle Unit, Stanmore, United Kingdom; 3 Northumbria NHS Healthcare Trust, North Tyneside General Hospital, North Shields, United Kingdom; 4 Department of Orthopaedics & Musculoskeletal Science, University College London, London, United Kingdom

**Keywords:** impaction bone grafting, ankle fusion, hindfoot fusion, tibiotalocalcaneal fusion, morselized, femoral head allograft, large bone defect, outcome studies, nonunion

## Abstract

**Background:**

Ankle and hindfoot fusion in the presence of large bony defects represents a challenging problem. The purpose of this study was to evaluate outcomes of patients who underwent ankle-hindfoot fusions with impaction bone grafting (IBG) with morselized femoral head allograft to fill large bony void defects.

**Methods:**

This was a 3-center, retrospective review of a consecutive series of 49 patients undergoing ankle or hindfoot fusions with femoral head IBG for filling large bony defects. Union was assessed clinically and radiologically with radiography or computed tomography. Graft stability/collapse was identified on radiographs as loss of graft height across the fusion interface. Indications included 35 failed total ankle arthroplasty, talar osteonecrosis and collapse (7 patients), failed ankle fusion (4 patients), trauma with bone loss or fracture nonunion (1 patients), and other (2 patients). Tibiotalocalcaneal (TTC) fusion was performed in 36 (73%) patients and ankle (TT) fusion in 13 (27%).

**Results:**

Mean age was 59.3 (19-78) years. Mean follow-up was 22.9 ± 8.3 months. Eighteen percent were smokers. Mean depth of the bone defect was 35.2 ±8.7 mm, and mean volume of the defect was 62.2 ±5.8 cm^3^. Symptomatic nonunion rate was 14% (7/49). The mean time to radiologic union was 7.6 ±3.2 months. Complete radiologic union rate occurred in 73% (36/49). Eight TTC fusion patients (22.2%) united at the tibiotalar joint but not at the subtalar joint, of which 6 were asymptomatic. There was no graft collapse, even in patients developing nonunion, with all patients maintaining bone incorporation and leg length.

**Conclusion:**

Impaction of morselized femoral head allograft can fill large bony voids around the ankle or hindfoot during fusion, with rapid graft incorporation and no graft collapse despite early loading. This technique offers satisfactory and comparable union outcomes without limb shortening or expensive custom 3D-printed metal cages.

## Graphical abstract

**Graphical Abstract:**
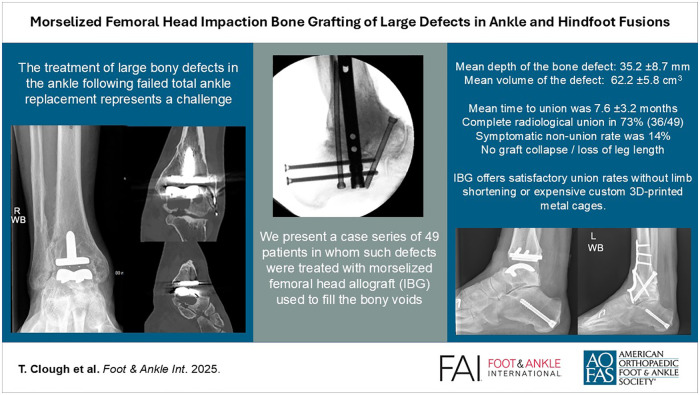
This is a visual representation of the abstract.

**Level of Evidence:** Level IV, retrospective case series

## Introduction

Ankle and hindfoot fusion in the presence of large bony voids or defects presents a challenge. These defects are commonly encountered following failed total ankle arthroplasty (TAA), talus avascular necrosis (AVN), fusion nonunion, fracture, and erosive arthritis.^9,17,20^ The number of TAA is increasing (around 1000 cases per year in the United Kingdom), with a corresponding increase in cases requiring revision.^28^ With revision arthroplasty rates roughly 10% to 15% at 10 years,^5,28^ it is estimated that 5000 failed TAA will require treatment in the United Kingdom alone over the next 2 decades.^22^

Options for failed TAA include revision TAA or conversion to fusion. In a recent meta-analysis, 26.9% of revision TAAs required further surgical intervention, and 14.4% required a rerevisional procedure. When revision of a failed TAA to another ankle joint replacement is not appropriate (in cases of significant bone loss), salvage fusion options include tibiotalar (TT) and tibiotalocalcaneal (TTC) fusion. One of the main challenges with these fusions is the management of the large bone void following removal of the TAA. Current accepted options include acute shortening and fusion,^3^ bulk structural allograft,^6-9,16,23,30^ noncustom metal cages,^3^ and 3D-printed custom metal cages.^3,9,17,20,23,29,34^

Direct fusion without graft can lead to significant limb shortening, poor patient satisfaction, and considerable gait alteration.^3,40^ Structural bone graft aims to maintain limb length; options include iliac crest autograft,^12^ fibular strut grafts,^35^ or bulk allograft. Harvesting iliac crest autograft is associated with significant donor site morbidity and is insufficient for filling large defects. Structural bulk allograft with fresh frozen femoral head has reported union rates ranging from 48% to 90.7% (with a meta-analysis reporting a union rate of 67.4%),^8,23,33,38^ graft resorption and collapse of up to 57%,^26,38^ and an infection and amputation rate of up to 19%.^8,23^

Noncustom trabecular metal cages have inconsistent satisfaction rates, and reported implant incorporation is poor at up to 50%.^4,20^ 3D-printed custom metal cages have gained recent popularity; however, reports suggest variable union rates between 74% and 90%,^2,10,31^ amputation rates of 5% to 14%,^2,6,31^ fractures,^1^ and costs of up to $20,000 per custom implant.^32^ Furthermore, most of the reports are case reports at present.

We report on a “new” technique using morselized impaction bone grafting (IBG) of femoral head allograft to fill the void, maintain limb length, and achieve union. Morselized impaction bone grafting has been successfully used since 1984 by hip and knee surgeons as an alternative of metal trusses and cages in revision arthroplasty surgery.^19,30,37,39,41^ However, to our knowledge, its use has not yet been reported in the foot and ankle literature.

The principal aims of this study were to report our clinical and radiologic union rate in a consecutive series of 49 patients that were treated with IBG of morselized femoral head allograft to fill large hindfoot bone defects. Secondary aims were to assess graft stability/collapse, and to report on complications, reoperations and postoperative patient-reported outcome measures.

## Methods

We conducted a retrospective review of all patients undergoing ankle and/or hindfoot fusions with IBG of morselized femoral head allograft to fill large bony void defects, with a minimum follow-up of 1 year. The cases were identified from the hospital *International Classification of Diseases* (*ICD*) codes. These procedures were carried out by the senior authors in 3 centers between May 2013 and March 2023.

**Figure 1.**
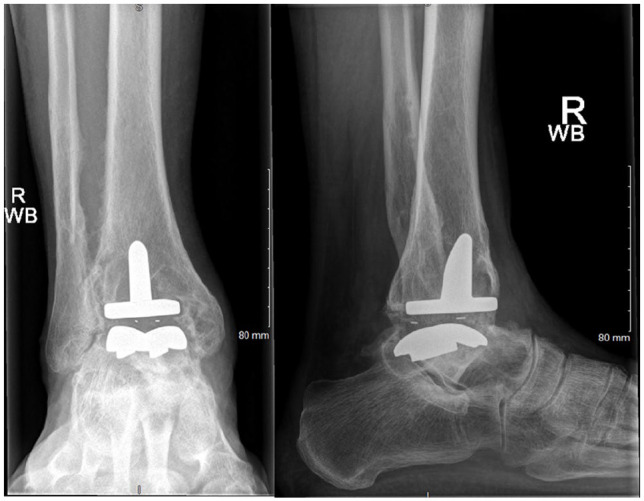
Preoperative radiographs of failed total ankle arthroplasty (Ankle Evolutive System) with large bony defect measuring >4 cm.

**Figure 2.**
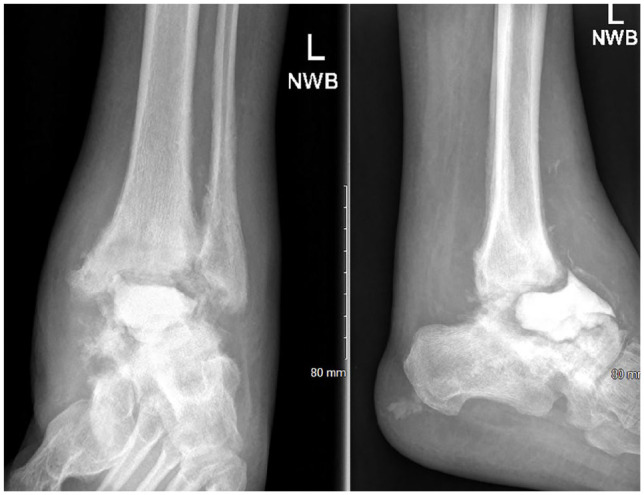
Preoperative radiographs of a patient with infected talar osteonecrosis with cement spacer in situ—bony defect measuring >4 cm.

The decision to proceed with IBG was based on the treating surgeon’s assessment of the imaging and anticipation of a large defect, >2 cm in any dimension, that would likely not be filled with autograft alone (Figures 1 and 2). Forty-nine consecutive patients were identified who fitted the inclusion criteria and had at least 12 months of clinical and radiographic follow-up. Patient demographics, preoperative diagnosis, comorbidities, indications for surgery, size of bone defects, surgical approach, fixation method, subsequent procedures, complications, and clinical and radiographic outcomes were recorded.

The primary outcome was union as demonstrated by radiography or computed tomography (CT) scanning. Union on radiograph was defined as bridging of 3 or 4 cortices on the anteroposterior and lateral views and was independently assessed by a consultant musculoskeletal radiologist. As this was a pragmatic clinical study, CT scanning was not routinely performed in all centers in the absence of clinical/radiologic concern, because of the inherent radiation doses required with CT. Where a CT was obtained, union was defined as bony bridging of >50% of the area of fusion across each surface, and no significant resorption of graft. Lack of union at 1 or more of the operated joints was categorized as nonunion. The clinical outcome of nonunion was based on the persistence of postoperative symptoms and ongoing limitation of function in the operated limb at final follow-up.

Secondary outcomes included graft stability/collapse, with stability defined as the lack of substantial change in hardware positioning and collapse as loss of graft height across the fusion interface as defined on radiographs or CT. Other secondary outcomes were complications, reoperations, and patient-reported outcome measures (PROMs). Patients completed a Manchester-Oxford Foot Questionnaire (MOXFQ) and EuroQol 5-Dimension, 5-Level tool (EQ-5D-5L) at 12 months postoperatively at all centers.

The MOXFQ is a validated PROM for foot and ankle surgery.^13-15^ The scores are categorized into 3 subscales: walking/standing, pain, and issues with social interaction, and from this, a MOXFQ summative index score is defined. The scores are reported on a scale from 0 to 100, where 100 denotes the worst score. The EQ-5D-5L is a tool designed to measure health-related quality of life and has been shown to be responsive in foot and ankle conditions.^18,21^ A score of 1.000 represents perfect health and a score of 0.000 represents a health state as bad as being dead.

### Surgical Procedure

Preoperatively all patients were assessed clinically to ensure sufficient vascularity to the limb and adequate skin integrity for wound closure. In the setting of infection, patients were discussed in a multidisciplinary meeting including surgeons, radiologists, bone infection specialists, and microbiologists.

The surgical technique used was dependent on the case. All procedures were performed in clean air theatres with laminar flow, double skin preparation, and care taken throughout for careful hemostasis. The procedures were carried out under tourniquet with antibiotic prophylaxis. At the time of explanation, multiple tissue samples were taken for histology and microbiology culture before delivery of antibiotics. An anterior approach was used to access the tibiotalar joint. For the cases of TTC fusion, a separate sinus tarsi approach was performed to prepare the subtalar joint. We aimed to keep both the medial malleolus and fibula intact, which act as “sides” to the contained void, thus allowing impaction packing of the morselized bone graft. In the cases where infection was a concern, these were performed as a 2-stage technique, with initial removal of the hardware, debridement, biopsy, and insertion of a cement spacer (Figure 2), followed by later removal of the cement spacer, void filling with IBG technique, and fixation fusion. Where there were large cysts, any membrane was removed, and the cysts were curetted until bleeding bone was noted to allow for graft integration. The surfaces for fusion were fenestrated with a small osteotome.

A fresh frozen femoral head was thawed in warm saline. This was debrided of cartilage and cortical bone. A hand turned bone mill was used to achieve small uniform bone graft chips. Bone marrow aspirate, harvested via a Jamshidi needle from the patient’s iliac crest, was mixed with the small bone graft chips. The graft was packed into the void/defect initially with firm thumb pressure and then finished with a surgical punch, such that it was under firm pressure in the contained defect. The authors noted the back of the ankle void defect had a firm fibrous membrane/capsule. The sides were contained by the fibular laterally and the medial malleolus medially. The anterior ankle also had a firm fibrous capsular layer that could be closed to contain the packed cancellous chips under pressure. A large volume of cancellous chips was required, packed under pressure, to fill most defects.

**Figure 3.**
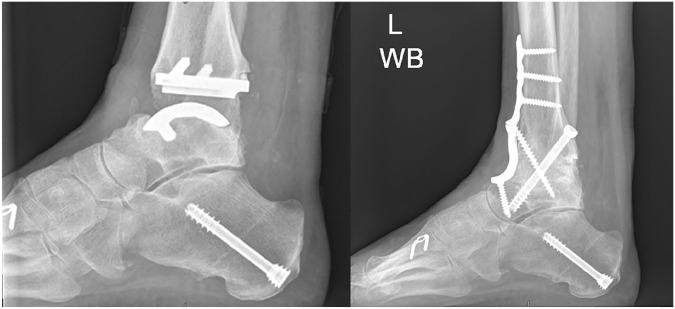
Pre- and postoperative lateral radiographs demonstrating tibiotalar fusion using an anterior ankle approach + screws. Preoperatively there was collapse of the talar dome with cystic change in both the tibia and talus. Radiographs confirm solid graft incorporation with no loss of height.

**Figure 4.**
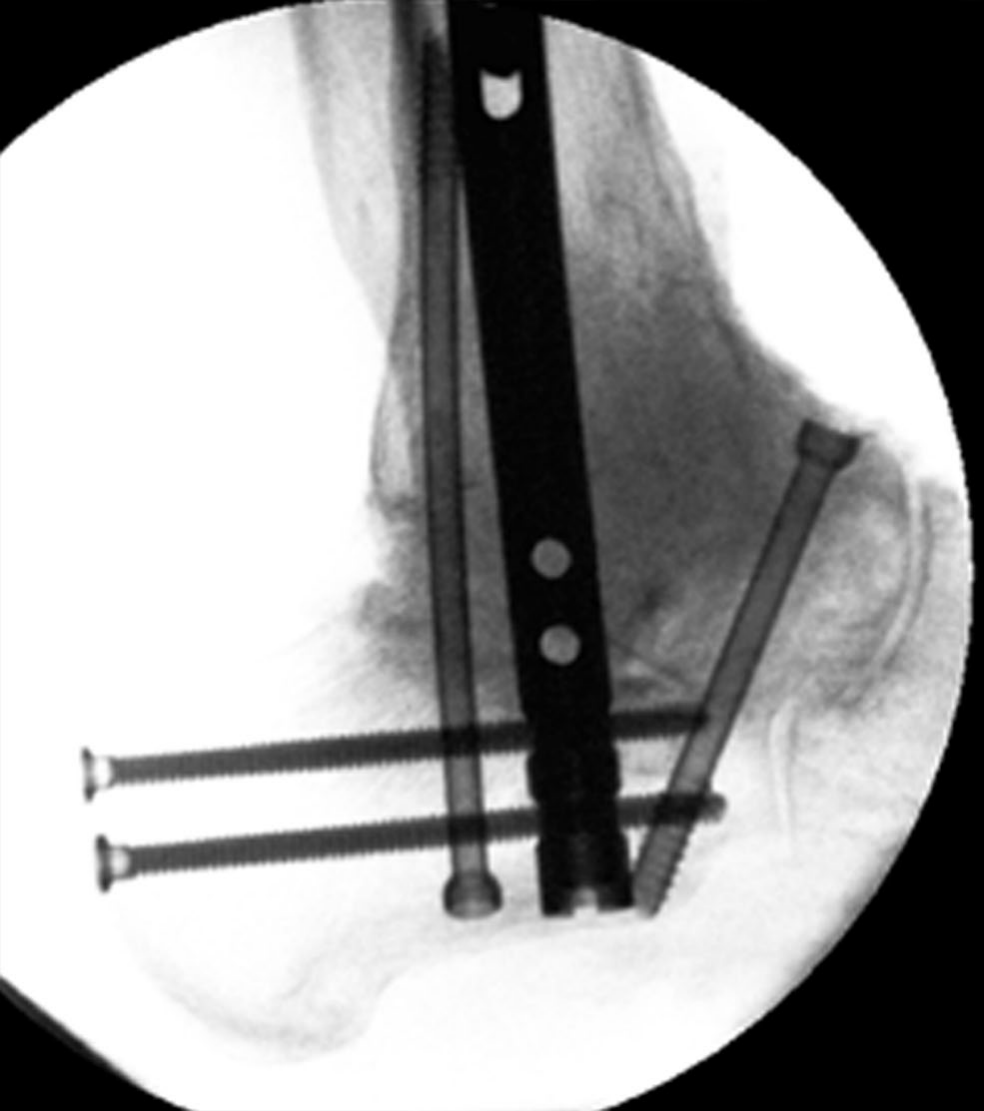
Intraoperative lateral radiographs of the case from Figure 1 showing the appearance on intraoperative imaging and the extent of bone graft impaction through the talus and tibia. In this case, an extra screw was used to secure the talar head to the construct as the talar head had poor bone stock.

**Figure 5.**
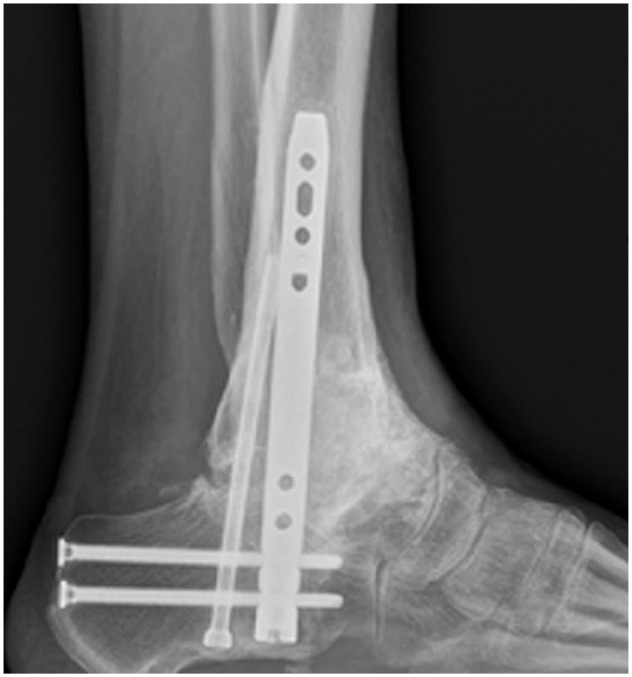
Postoperative lateral radiographs of the case from Figures 1 and 4 showing the extent of solid consolidation of all bone graft. The extra screw in the anterior talus had been removed alongside shaving down of anterior bone.

**Figure 6.**
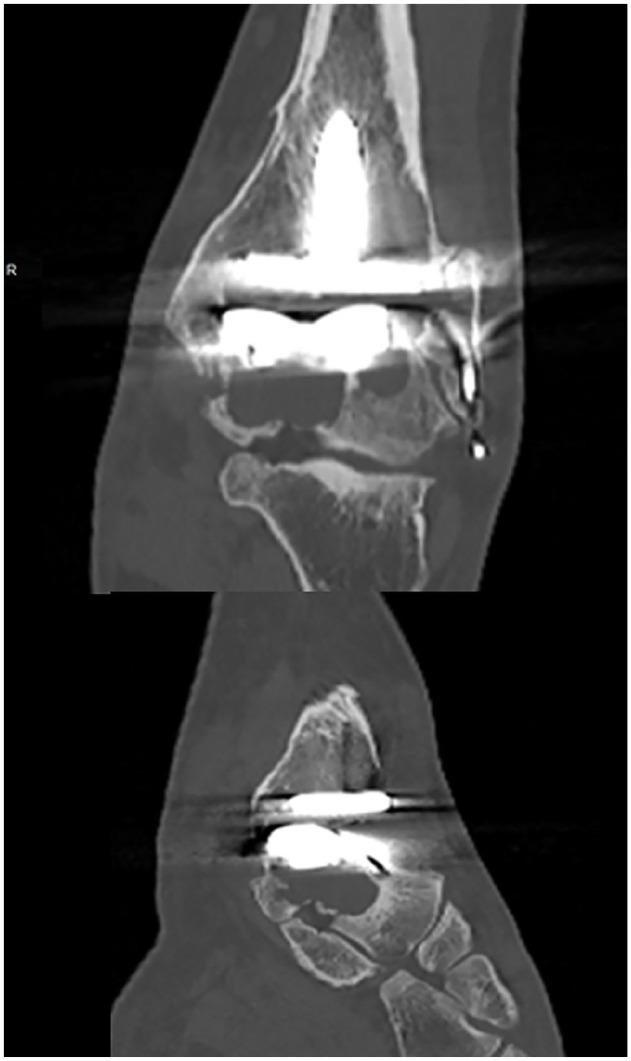
Preoperative coronal and sagittal computed tomographic slices of the case in Figure 7. In this case there were large talar cysts throughout the height of the talus and involving the anterior plafond.

**Figure 7.**
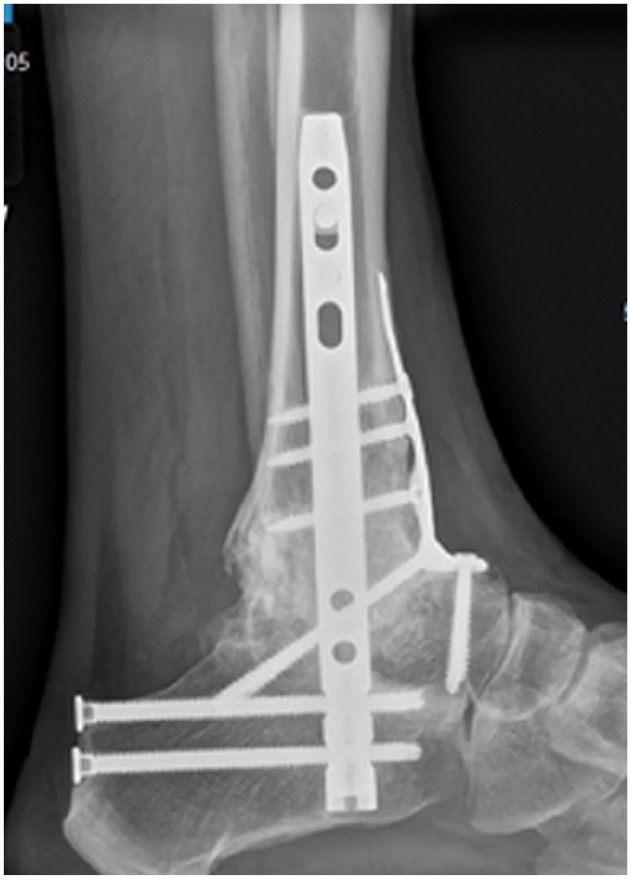
Postoperative lateral radiograph of the case in Figure 6 demonstrating tibiotalocalcaneal (TTC) fusion with intramedullary nail and anterior fusion plate using a combined anterior ankle and sinus tarsi approach. Radiographs confirm solid graft incorporation with no loss of height.

The fixation construct was dependent on the case and joints needing to be stabilized. For ankle fusions alone, an anterior plate, screws, or a combination of both were used (Figure 3). For TTC fusions, a hindfoot nail was used mainly (Figures 4 and 5), but, in some cases, both an anterior ankle fusion plate and a hindfoot nail were implanted, particularly where there was concern about bone quality, or there was a dual anterior and sinus tarsi approach, where the anterior plate could act to further hold the IBG in place (Figures 6 and 7). A nail-and-plate construct was also used in some cases where there was a free-floating talar head fragment after debridement, in order to gain some stability of the head fragment to the hindfoot fusion mass. Adequate compression was achieved across the fusion site regardless of the construct and was an essential part of the procedure.

The postoperative regimen consisted of 12 weeks in a below-knee plaster cast or surgical boot support, the initial 4 weeks of which were nonweightbearing, and all patients were fully weightbearing in a cast by 8 weeks. A wound check and removal of sutures was performed at 2-3 weeks postoperatively. Assessment of bony union was made clinically and radiologically from 12 weeks.

### Data Analysis

Statistical analysis was conducted using Medcalc Statistical Software, version 19.2 (MedCalc Software Ltd, Ostend, Belgium). Descriptive statistics were computed for all variables presented as mean values accompanied by their SDs.

## Results

The mean age of the patients in this study was 59.3 ± 14.7 years (range 19-78 years). There were 33 males and 16 females. Surgery was performed on the right limb in 23 and the left limb in 26 patients. Nine patients (18%) were smokers. Full patient demographics are listed in Table 1. Failed TAA was the most common indication with 35 (71%) patients. Other indications are listed in Table 2. The bony defect had a mean depth of 35 ± 8.7 mm (maximum void height of 49 mm) and a mean maximal estimated volume of 62.2 ± 5.8 cm^3^. The procedures carried out are summarized along with their respective union and complication rates in Table 2. Implants used were a hindfoot intramedullary nail in 30 cases (61%), intramedullary nail and anterior ankle plate combination in 6 cases (12%), anterior ankle plate in 9 cases (18%), screws and anterior plate combination in 2 cases (4%), and screws alone in 2 cases (4%) (Table 3).

**Table 1.** Demographic Data for Patient Cohort (49 Patients).

	Value^a^
Age, y, mean (range)	59.3 (19-78)
Sex	
Female	16
Male	33
BMI, mean (SD)	31.2 (7.2)
Diabetes	
Yes	5
No	44
RA/steroid use	
Yes	8
No	41
Neuropathy	
Yes	4
No	45
Smoking	
Yes	9
No	40
Prior limb infection	
Yes	6
No	43
Laterality	
Left	26
Right	23
Follow-up time, mo, mean (SD)	22.9 (8.3)
Depth defect, mm, mean (SD)	35.2 (8.7)
Volume defect, cm^3^, mean (SD)	62.2 (5.8)

Abbreviations: BMI, body mass index; RA, rheumatoid arthritis.

a Unless otherwise noted, values are n (%).

**Table 2.** Diagnosis, Fixation Constructs, Nonunion, and Complications.

Diagnosis	No. of Cases	Fixation	No. of Cases	Nonunion	Complications
Failed TAA	35				
Aseptic loosening	30	Hindfoot nail	18	NU: 1 STJ only: 4 (3 asymptomatic)	Delayed wound healing: 3 Late nail breakage: 1
		Hindfoot nail + anterior ankle plate	6	NU: 1 STJ only: 1 (asymptomatic)	Delayed wound healing: 1
		Anterior ankle plate + screws	2	0	
		Anterior ankle plate	4	0	Stress fracture:1
Infection	5	Hindfoot nail	4	NU: 1 STJ only: 1	Delay wound healing: 1
		Anterior ankle plate	1	0	
Talar osteonecrosis/collapse	7	Hindfoot nail	6	NU: 1 STJ only: 1 (asymptomatic)	CRPS: 1
		Screws only	1	NU: 1	
Failed previous ankle fusion	4	Anterior ankle plate	3	0	Stress fracture: 1
		Hindfoot nail	1	STJ only: 1 (asymptomatic)	
Fracture nonunion				0	
Talus	1	Hindfoot nail	1	0	
HMN	1	Anterior ankle plate	1	0	
Defect post GCT excision	1	Screws only	1	0	

Abbreviations: CRPS, Chronic Regional Pain Syndrome; GCT, giant cell tumor; HMN, hereditary motor-neuropathy; NU, nonunion of all joints; STJ, subtalar joint; TAA, total ankle arthroplasty; TT, tibiotalar; TTC, tibiotalar-calcaneal.

**Table 3.** Secondary Outcome Measures and Reoperation Rates.

	n (%) or mean (range)
Graft collapse/resorption	0
Reoperation other than revision	7 (14)
Amputation	1 (2)
MOXFQ score	47.9 (11-94)
EQ-5D-5L score	0.584 (0.182-0.861)
VAS score	4.2/10 (0-9)

Abbreviations: MOXFQ, Manchester-Oxford Foot Questionnaire; EQ-5D-5L, EuroQol 5-dimension, 5-level tool; VAS, visual analog scale

At a mean follow-up of 22.9 ± 8.3 months, the combined symptomatic nonunion rate was 14% (7 patients). Mean time to radiologic union was 7.6 ±3.2 months. Union was confirmed based on radiographs in 29 patients and CT in 20 patients. Complete radiologic union rate was 73% (36 of 49 patients). Eight TTC fusion patients (22.2%) united at the tibiotalar joint but not at the subtalar joint, of which only 2 were symptomatic. Both symptomatic patients (4%) underwent attempted revision fusion, with 1 uniting.

**Figure 8.**
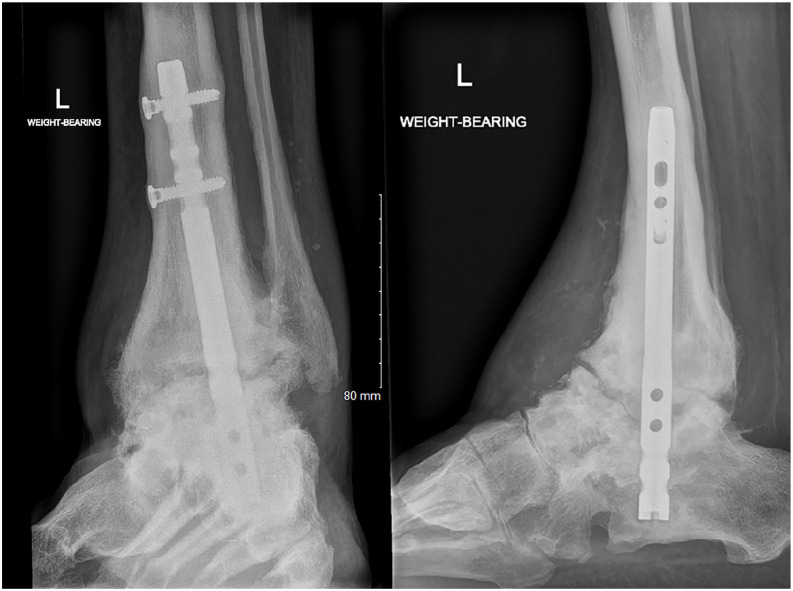
Postoperative radiographs of tibiotalocalcaneal nonunion with restoration of bone stock. (Follow-up imaging of Figure 2.)

Reoperation, other than revision, occurred in 7 patients (14%); 6 had removal of symptomatic metalwork and 1 patient with asymptomatic subtalar nonunion had late metalwork failure at 78 months, requiring removal. Two patients (4%) developed a stress fracture above the anterior ankle fusion plate that healed with nonoperative measures. There was no bone graft collapse, with all patients maintaining length across the graft void. Even in patients with established nonunion we observed graft incorporation and restoration of bone stock despite the lack of definitive union (Figure 8). The 6 asymptomatic nonunion patients (12%) were satisfied with the procedure and were therefore treated conservatively. No patients developed deep infection. One patient required a below-knee amputation for persistent nonunion (2%) (Table 3).

We obtained an 84% return rate for our postoperative PROMs. The average postoperative MOXFQ score was 47.9 (range 11-94), the average visual analog pain score was 4.2 of 10 (range 0-9), and the average postoperative EQ-5D-5L score was 0.584 (0.182-0.861).

## Discussion

In this series we demonstrate a limb length–preserving, salvage procedure for these complex procedures. There are several key features and advantages to the technique of IBG. Although in isolation, morselized graft has no inherent strength, when packed into a contained defect or void, under load, it acts like a liquid, resisting pressure with little deformation.^36^ Consequently, the construct allows for early weightbearing without collapse or deformation, which in turn promotes rapid bone incorporation. Previous studies have demonstrated that once IBG incorporates, it is difficult to differentiate between donor and host bone histologically.^27^ It therefore effectively restores bone stock. Our grafts incorporated within an average of 7.6 months, with preservation of height, even with a maximal graft height of 49 mm. We saw no late collapse or graft fracture, even out to 7 years, which has been reported with bulk (structural) allograft^26^ and synthetic cages.^38^ Finally, morselizing the graft makes it easily amenable to packing and allows precise filling of defects of any shape without the need to resect healthy local bone.

Even with an early weightbearing protocol, there was no bone graft collapse with all patients maintaining length across the graft void. The authors stress that this can only happen if the defect is contained, thus preventing escape of the impaction chippings. Our surgical approach was anterior, and we aimed to keep both the medial malleolus and fibula intact, which acted as “sides” to the contained void. Where the fibula had been already harvested, the authors anticipate that with a revision lateral approach, the void could still be “contained” with use of a lateral plate, thus making the technique viable in these instances. In those patients who developed an established nonunion, we still observed graft incorporation and restoration of bone stock despite the lack of definitive union (Figure 8). This contrasts with reports of up to 57% graft resorption with bulk femoral head allograft at an average 30-month follow-up.^1^ We consider this to be an advantage over bulk allograft as it facilitates further revision options and limb salvage.

The mean age of our patients was 59.3 years, comparable to the meta-analysis of 175 patients by Cifaldi et al^9^ of mean age 60.5 years. Our indications are similar to previous authors, with failed TAA and talar osteonecrosis our most common indications.^9,20^ Most of our patients (73%) had a TTC fusion, which is the most frequently performed salvage procedure in this situation, but 27% had a TT fusion. Only 3 of the 11 studies that examined ankle and hindfoot fusion in the presence of a large bone defect included cases of isolated tibiotalar fusions.^7,9,16^ The most common fixation constructs in our series was intramedullary nail (61%), followed by an anterior ankle fusion plate (18%), which is similar to the findings from the pooled data of 11 studies (89% and 8%, respectively).^9^ Our patient cohort is therefore comparable to that of previous studies, although 18% of our cohort were smokers.

The radiologic union rate for TTC fusions was 67%, with most (8 of 12) failing at the subtalar joint. Of those failing at the subtalar joint, 75% (6 of 8) were clinically asymptomatic. Radiologic union rate for revision ankle fusion was 92%. Structural allograft with fresh frozen femoral head has reported union rates ranging from 43% to 75%.^1,8,23,33^ A meta-analysis pooling 175 patients found a similar overall union rate of 67.4%.^9^ The variation in the reported union rates in the literature could be the result of the disparity between the different series regarding their patients’ characteristics (age, smoking, and diabetes), sample size, indications for revision, size of bony defects, occurrence of complications, constructs used, use of additional bone graft or biologic products, methods of assessing fusion, and duration of follow up.^9,20^ In our study, 6 of our 13 nonunion patients (46.2%) were asymptomatic and were satisfied with the procedure, requiring no further surgery. Several authors have observed similar experiences. Jeng et al^23^ reported that 7 of their 16 nonunion patients achieved a stable, asymptomatic nonunion. Bussewitz et al^8^ stated that 9 of 13 nonunion patients had a stable, painless limb.

When comparing radiologic union rates across diagnosis, we had a 77% union rate for aseptic loosening of failed TAA, a 60% union rate for infected failed TAA, a 57% union rate for talar osteonecrosis and collapse, and a 75% union rate for fusion of failed previous ankle fusion (Table 3).

In our series, 7 patients (14.3%) had a symptomatic nonunion and a further 7 patients (14.3%) underwent reoperation other than revision, which compares favorably both to Jennison’s analysis of the UK National Joint Registry (NJR) data of 18.3% patients requiring rerevisional fusion surgery after failure of fusion following failed TAA and 24.4% patients undergoing reoperation other than revision,^25^ and his systematic review and meta-analysis reporting 13% nonunion rates (from 23 papers reviewing conversion of failed TAA to fusion).^24^ Our overall complication rate was relatively low (18%) with no cases of deep infection. This compares to a complication rate of 26.6% with use of bulk structural allograft^9^ and a reported infection rate 14.3% with cages.^6^ Only 1 of our patients (2%) required an amputation. Jeng et al^23^ reported an amputation rate of 19% following TTC fusion with bulk structural allograft in 32 patients, whereas Abar et al^2^ reported an amputation rate of 13% with 3D custom cages in 39 patients. The reason for our low infection rate is likely multifactorial, but contributing factors may include fastidiousness in skin preparation, wound handling, hemostasis and wound closure. We also staged procedures where infection was initially present to ensure its eradication prior to surgery. Infected cases were managed in a multidisciplinary setting, which helped target appropriate antibiotic therapy.

The cost of 3D-printed titanium custom implants has become a concern for most centers managing this clinical problem, with average 3D-printed titanium implant costs alone ranging from £13 000 to $20 000.^32^ By contrast, the costs of a femoral head allograft is around $950. There is therefore a significant cost benefit to this technique.

Although we describe the benefits of impaction bone grafting, it is worth mentioning some other potential options for treatment. These include direct fusion without graft, which has high reported union rates (95.6% for tibiotalar, and 87.5% for subtalar),^3^ but this results in acute shortening. Although this may be treated with proximal lengthening, there are few studies reporting outcomes of this, and this would result in potentially prolonged healing times. Bone transport with ankle arthrodesis has also been described for management of large bony defects.^24,25,34^ These report a union rate of 85.7%-94% in the posttraumatic setting, although this may not be suitable for settings where the subtalar joint also needs to be fused, and there are no reported series to our knowledge for this indication. Tibial or femoral intramedullary reaming using a Reamer-Irrigator-Aspirator system may also be a potential source of autograft; however, the authors do not have experience with this for this indication and cannot surmise whether this would handle the same way as morselized femoral head allograft. It would also potentially violate another joint and may not be possible in the setting of a knee replacement.

This study has limitations. First, it is retrospective in nature, although for this consecutive series, no patient was lost to follow-up. It was not routine practice to use CT scanning to assess for radiologic union. Because of the inherent radiation doses associated with its use, CT was used based only on clinical need and concern for nonunion. Although it is recognized that CT is more reliable than radiographs for assessing osseous union,^11^ most of the reported series in the literature use radiographs to determine union rates.^9^ Although the cohort in this study is larger than most reporting this issue, the heterogenous indications make the study underpowered to detect further associations between preoperative variables and the surgical outcome. Also, this is a clinical outcome study of a heterogenous group of consecutive series of patients undergoing a single technique of impaction bone grafting to fill large bone voids and lacks a comparative group, though the authors compare their results of this ‘new’ technique to those from the literature of alternative established techniques. Finally, we did not have preoperative PROMs for our patients, and so cannot establish how much of an improvement patients experienced with this technique and surgery. However, our reported PROMs can serve as a baseline for future and further research.

## Conclusion

Impaction of morselized femoral head allograft can be used to fill large bony voids around the ankle and hindfoot when undertaking fusion, with relatively rapid graft incorporation and no graft collapse, with the advantage of early weightbearing. Our experience suggests that this innovative technique offers satisfactory and comparable clinical outcomes for these difficult clinical problems, restores bone stock without the need for limb shortening or expensive synthetic cages, and avoids the risk of late graft collapse which can be seen with bulk structural allograft. It also allows the filling of the irregular shaped voids that are common in this patient cohort without the need for further bony resection. Longer-term follow-up would be beneficial, and further comparative, prospective studies are required to determine the efficacy of this procedure compared to other salvage techniques.

## Supplemental Material

sj-pdf-1-fai-10.1177_10711007241310411 – Supplemental material for Morselized Femoral Head Impaction Bone Grafting of Large Defects in Ankle and Hindfoot Fusions Supplemental material, sj-pdf-1-fai-10.1177_10711007241310411 for Morselized Femoral Head Impaction Bone Grafting of Large Defects in Ankle and Hindfoot Fusions by Tim Clough, Bakur Jamjoom, Naeem Jagani, Jared Quarcoopome, Rajesh Kakwani, David Townshend, Nicholas Cullen, Shelain Patel, Karan Malhotra and Matthew Welck in Foot & Ankle International
